# CD133 expression is not an independent prognostic factor in stage II and III colorectal cancer but may predict the better outcome in patients with adjuvant therapy

**DOI:** 10.1186/1471-2407-13-166

**Published:** 2013-03-28

**Authors:** Khalilullah Mia-Jan, So Young Jung, Ik-Yong Kim, Sung Soo Oh, EunHee Choi, Sei Jin Chang, Tae Young Kang, Mee-Yon Cho

**Affiliations:** 1 Department of Pathology, Yonsei University Wonju College of Medicine, Wonju, South Korea; 2 Department of Surgery, Yonsei University Wonju College of Medicine, Wonju, South Korea; 3 Department of Occupational and Environmental Medicine, Yonsei University Wonju College of Medicine, Wonju, Korea; 4 Institute of Lifestyle Medicine, Yonsei University Wonju College of Medicine, Wonju, Korea; 5 Preventive Medicine, Yonsei University Wonju College of Medicine, Wonju, Korea; 6 Institute of Genomic Cohort, Yonsei University Wonju College of Medicine, Wonju, Korea

**Keywords:** Cancer stem cell, CD133 protein, Human, Colorectal neoplasms, Immunohistochemistry, Chemoradiotherapy, Adjuvant, Prognosis

## Abstract

**Background:**

Cancer stem cells (CSCs) are notorious for their capacity of tumor progression, metastasis or resistance to chemo-radiotherapy. However, the undisputed role of cancer stem marker, CD133, in colorectal cancers (CRCs) is not clear yet.

**Methods:**

We assessed 271 surgically-resected stage II and III primary CRCs with (171) and without (100) adjuvant therapy after surgery. CD133 expression was analyzed by immunohistochemical (IHC) staining and real-time RT-PCR. CD133 promoter methylation was quantified by pyrosequencing.

**Results:**

The CD133 IHC expression was significantly correlated with mRNA expression (p=0.0257) and inversely correlated with the promoter methylation (p=0.0001). CD133 was expressed more frequently in rectal cancer (p=0.0035), and in moderately differentiated tumors (p=0.0378). In survival analysis, CD133 expression was not significantly correlated with overall survival (OS) (p=0.9689) as well as disease-free survival (DFS) (p=0.2103). However, CD133+ tumors were significantly associated with better OS in patients with adjuvant therapy compared to those without adjuvant therapy (p<0.0001, HR 0.125, 95% CI 0.052-0.299). But the patients with CD133- tumors did not show any significant difference of survival according to adjuvant therapy (p=0.055, HR 0.500, 95% CI 0.247-1.015).

**Conclusions:**

In stage II and III CRCs, CD133 IHC expression may signify the benefit for adjuvant therapy although it is not an independent prognostic factor.

## Background

Cancer stem cells (CSC)s are undifferentiated cells that expand their colony through asymmetric cell division, the result of which is two daughter cell population, one being similar to the mother cells, retaining stem cell properties, while the other one is committed to undergo a specified differentiation [1]. CSCs have been isolated from many hematologic and solid tumors including colorectal cancers (CRC)s and they have been defined to have the capacity of self-renewal and multipotency [2] and ability to maintain the stem cell pool and most elements of the tumor for unlimited time period [3,4] being responsible for tumor initiation and progression [5], resistance against chemo-radiotherapy, and relapse after initial eradication [6].

Different markers have been found to be expressed on the surface of CSCs, out of which CD133 has retained much attention and importance. The CD133+ population exists among cancer initiating cells in many tissues, including colon [7], breast [8], lung [9], stomach [10], liver [11], gallbladder [12],prostate [13], endometrial [14], pancreatic carcinomas [15], leukemia [16], glioma [17], and medulloblastoma [18].

CD133 or Prominin-1 is a pentaspan transmembrane glycoprotein [19], whose gene is located on chromosome 4p15.32. CD133 comprises five transmembrane domains and two large glycosylated extracellular loops [20]. Three of the five promoters responsible for CD133 transcription are located in a CpG island [21]. Thus, epigenetic factors can complicate the regulation of CD133 gene transcription [22]. DNA hypomethylation is accounted as an important determinant of CD133 expression [23]; however, yet the regulatory mechanism of CD133 gene transcription is not utterly understood.

CD133 expression is reported to be indicative of a resistance phenotype [24], poor prognosis [25], and are believed to mediate cancer relapse after chemotherapy [26] and lower level of CD133 mRNA expression are documented to be associated with a longer relapse-free interval and overall survival (OS) in colon cancer [27]. Controversially, it was recently shown that CD133+ cells are not more resistant to chemotherapy than CD133− cells [28]. On the other hand, the evidence provided by Huang, E. H. et al., shows that nude mice injected with CD133 negative colon cancer cells developed cancer [29]. Moreover, Du. L. et al. have demonstrated that knock-down of CD133 does not compromise the tumor-initiating capabilities of colon cancer cells, questioning a functional role of this molecule for the colon cancer stem cells [30].

In this study, we evaluated CD133 expression by immunohistochemical (IHC) stains and real-time RT-PCR in CRCs. In addition, promoter methylation status was analyzed by pyrosequencing. We further analyzed the prognostic significance of CD133 expression in CRCs and correlation of promoter methylation status with IHC and mRNA expression.

## Methods

### Patients and tissue samples

This retrospective study included the patients who had surgically resected stage II and III CRC and available follow-up information from January 2000 to December 2006. For the comparison, we divided the patients according to the tumor location and either receiving adjuvant treatment or no adjuvant treatment. The patients receiving preoperative chemotherapy or radiotherapy were excluded. Clinicopathologic data for parameters such as patient’s age, gender, tumor location, invasion depth, histologic differentiation, and lymph node metastasis were collected from the pathology report. For the comparison, we also performed CD133 IHC staining on non-neoplastic gastric mucosa and pancreatic parenchyma.

### Ethics approval

The study has been approved by the Institutional Ethic Committee of Yonsei University, Wonju College of Medicine (YWMR-12-4-031) and has been carried out in compliance with the guidelines of the Declaration of Helsinki.

### Adjuvant chemotherapy and follow-up

Patients were planned to receive 5^th^ cycles to 12 cycles of adjuvant FOLFOX chemotherapy within a six-month period. The patients received a 2-hour infusion of 85 mg of oxaliplatin per square meter on day 1, in addition to the standard LV5FU2 regimen (FOLFOX4) or the simplified LV5FU2 regimen (modified FOLFOX6). After surgery, tumor recurrence was detected by physical examination, serum CEA antigen assay, and abdominal imaging every three to six months for 3 years, every six months for the following 2 years, and then annually. The duration of follow-up was defined as the time between surgery and disease recurrence (DFS), death (OS), or last hospital contact (scheduled follow-up or telephone contact). Maximum follow-up period was 108.3 months.

### Immunohistochemical analysis

Paraffin-embedded tissue sections from well representative blocks were deparaffinized with xylene and then rehydrated through graded alcohol solutions. Antigen retrieval (according to manufacturer’s instructions) consisted of slide warm-up to 75°C and incubation (4 min), applying cell conditioning solution #2 (60 min) (Ventana medical system, Roche, Tucson, USA). Endogenous peroxidase was blocked by applying UV inhibitor (4 min). The slides were washed with reaction buffer. The UltraView Universal DAB Detection Kit was used for IHC staining. The steps are briefly described as following. The primary antibody (CD133/1 (AC133) pure, Human, MACS, Miltenyi Biotec, CA, USA) was applied and incubated for 2 hrs at a 1:100 dilution in Ventana machine (BenchmarkXT, Ventana medical system, Roche, Tucson, USA). Slides were then rinsed with reaction buffer and added one drop of HRP UNIV MULT, DAB and DAB H2O2 (Ventana medical system, Roche, Tucson, USA) (8 min each), consecutively with intermittent rinsing with reaction buffer. Slides were then treated with one drop of COPPER (4 min) before counterstaining with hematoxylin (4 min), followed by bluing agent and finally rinsed with reaction buffer.

The IHC staining was scored as 0 when there was no expression at all, 1+ when the expression of CD133 was detected in 1-10% of the whole tumor area, 2+ and 3+ when it was expressed in 11-50% and 51-100% of the tumor area, respectively. Tumors with CD133 expression on over 10% of whole tumor area were considered as CD133 positive.

The IHC staining results were evaluated independently by two pathologists blinded to the patients’ clinical and pathologic information. Discrepancies between the pathologists were resolved by consensus.

### RNA Extraction and cDNA synthesis

Fresh frozen tissues after surgery were available for 75 out of 271 cases.

The total RNA was extracted from 20 mg colorectal frozen tissue, using RNeasy plus Mini kit (QIAGEN Hilden, Germany) according to manufacturer’s protocol and Quantitect Reverse Transcription kit (QIAGEN Hilden, Germany) was used for cDNA synthesis from 500ng of total RNA.

### Quantitative RT-PCR

Real-time RT-PCR was performed [as described elsewhere [31] in 384 well PCR plates containing the Fast SYBR Green Master Mix (Applied Biosystems, California, USA), cDNA template, CD133 RT sense primer (5'-CTGGGGCTGCTGT TTATTATTCTG-3'), and CD133 RT antisense primer (5'-ACGCCTTGTCCTTGGTAGTGTTG-3') in a final volume of 10 μL. Each primer/cDNA set was set up in triplicate. Real-time PCR reactions in a 7900HT Fast Real-Time PCR System (Applied Biosystems) were initiated by heating to 50°C for 2 min and then to 95°C for 10 min, followed by 40 cycles of 95°C (15 s), and 60°C (60 s). The relative quantification of gene expression was performed using the ΔΔCt method.

### Bisulfite conversion and pyrosequencing analysis of DNA methylation

We extracted DNA from microdissected sample using DNeasy Blood and Tissue kit (QIAGEN, Hilden, Germany) according to manufacturer’s instructions. Genomic DNA was modified with sodium bisulfite using an EpiTect® Bisulfite kit (QIAGEN Hilden, Germany) according to manufacturer’s instruction. Methylation status of CD133 was assessed using pyrosequencing-based methylation analysis. We evaluated the methylation status of CpG sites in promoter P2 and exon 1B, as these sites have previously shown correlation with CD133 gene transcript [31]. All primers for pyrosequencing were designed with Pyrosequencing Assay Design (QIAGEN, Hilden, Germany). Bisulfite-treated genomic DNA was used as a template in subsequent polymerase chain reactions. For each gene, a 30 ul PCR was carried out with HotStarTaqPlus Master Mix (QIAGENE, Hilden, Germany) to label bisulfite converted DNA with biotinylated primers (CD133 promoter site: Forward 5’-GGAGTAGGGATATGGGGGTATAAA-3’, Reverse primer 5’- AAACACCCCAATTCTCCATCT-3’). The PCR conditions included denaturation 94°C (30 s), annealing 54°C (30 s), extension 72°C (30 s) and 40 cycle. After PCR, the biotinylated strand was captured on streptavidin-coated beads (Amersham Bioscience, Uppsala, Sweden) and incubated with sequencing primers (5’- GGGATATGGGGGTATAAA-3’). The sequencing primer includes four methylation sites (GYGAGGTTATTTTTTYGYGTTYGTGGG). Pyrosequencing was performed with PSQ HS 24 Gold single-nucleotide polymorphism reagents on a PSQ HS 24 pyrosequencing machine (Biotage, Uppsala, Sweden).

### Statistical analysis

χ ^2^-test and Mantel-Haenszel test were used to analyze the categorical data. We used Pearson correlation to compare distributions of qualitative variables. Survival curve was estimated with the Kaplan-Meier method and compared using the log-rank test. Multivariate Cox proportional hazard regression model was used to estimate the hazard ratio (HR) and 95% confidence interval (CI) with adjustment for age and stage. Analyses were performed using SAS (Version 9.2, SAS Inc., North Carolina, USA). A value of p<0.05 was considered statistically significant.

## Results

### Patient characteristics

In this study, 161 male and 110 female patients were included in a randomized manner. Mean age for all 271 patients was 63.166 years (range 27–101). The characteristics of patients analyzed in this study according to tumor location and adjuvant therapy status is summarized in Tables 1 &2.

**Table 1:** The number of patients according to adjuvant therapy in colon and rectum*

**Location**	**Adjuvant therapy (stage II + stage III)**	**No adjuvant therapy (stage II + stage III)**	**Total (stage II + stage III)**
Colon	91 (40 + 51)	59 (38 + 21)	150 (78 +72)
Rectum	80 (23 + 57)	41 (21 + 20)	121 (44 + 77)
Total	171 (63 + 108)	100 (59 + 41)	271 (122 + 149)

*p=0.3337.

**Table 2:** The number of patients according differentiation in colon and rectum*

**Location**	**Well (%)**	**Moderate (%)**	**Poor (%)**	**Total (%)**
Colon	9 (6.00)	121 (80.66)	20 (13.33)	150 (100)
Rectum	7 (5.78)	104 (85.95)	10 (8.26)	121 (100)
Total	16 (5.90)	225 (83.02)	30 (11.07)	271 (100)

*p=0.4065.

### CD133 Immunohistochemical expression according to the clinicopathologic variables

**Figure 1:**
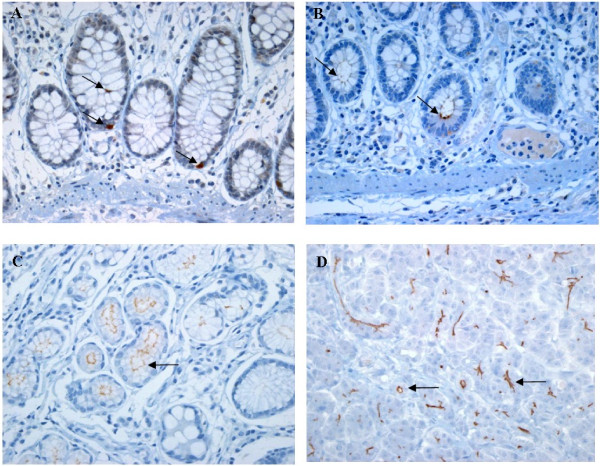
**Photomicrographs of CD 133 IHC expression in non-neoplastic colonic mucosa in comparison with the non-neoplastic glands of stomach and pancreas.** (**A**) CD133 positive cells are very rarely found in normal colon crypts and (**B**) along the luminal border of few crypts. (X 400 hematoxylin counterstained). In contrast to the colon, (**C**) the non-neoplastic pyloric glands and (**D**) pancreatic duct or acini show a distinct and diffuse staining in the luminal border. (X 200 hematoxylin counterstained).

A weak CD133 IHC expression in non-neoplastic colorectal mucosa around the tumor was noted in a few scattered cells (Figure 1A) and luminal border at the base of normal crypts (Figure 1B). On the contrary, we observed weak but frequent CD133 expression in non-neoplastic pyloric gland of stomach in some cases but not in fundic glands or mucus neck cells (Figure 1C). In pancreas, there are diffuse and strong CD133 expression in luminal border of non-neoplastic pancreatic duct as well as acini in all cases examined (Figure 1D).

**Figure 2:**
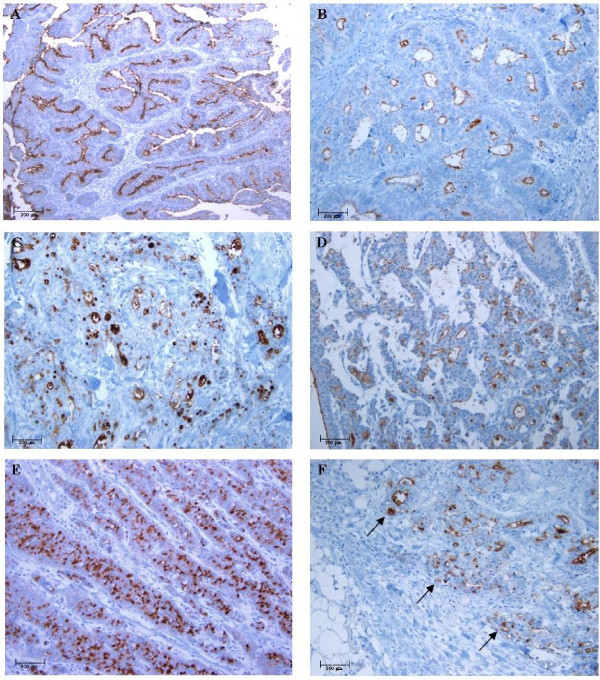
**The CD133 IHC expression according to the histologic differentiation of tumors.** CD133 is expressed along the glandular luminal side in (**A**) well-differentiated, (**B**) moderately differentiated, (**C**) poorly differentiated tumors, and (**D**) mucinous adenocarcinomas. Dot-like cytoplasmic staining is observed in poor histologic differentiation (**E**). CD133 IHC expression is also detected in cancer cells in invasive front (black arrows) (**F**). (X 100 hematoxylin counterstained).

In colorectal carcinoma, CD133 IHC expression was seen exclusively on the cell membrane at the glandular luminal surface of cancer glands in 192 of 271 tumors (Figure 2A-D &2F). Few tumors with poor differentiation (Figure 2C), tumor budding and mucinous adenocarcinomas (Figure 2D) showed focal CD133 expression in areas with abortive glands or intracytoplasmic luminal structure. Some tumors with poor histologic grade and mucinous adenocarcinomas showed dot-like cytoplasmic staining (Figure 2E). The intraglandular debris of shed tumor cells in some cases showed CD133 immunoreactivity, which were not taken into account. CD133 expression according to the clinicopathologic parameters are demonstrated in Table 3. In *χ*^2^- analysis and Mantel-Haenszel test, CD133 IHC expression was significantly different according to histologic differentiation (p=0.0378) and tumor location (colon vs. rectum) (p=0.0158, Table 3). The moderately differentiated tumors and rectal tumors showed more CD133 expression than others. There was no significant relationship between CD133 IHC expression and other clinicopathologic variables studied such as sex (p*=*0.8233*)*, pTNM stage (p=0.3598), invasion depth (p=0.160), and lymph node metastasis (p=0.346).

**Table 3:** CD133 expression according to the clinicopathologic parameters in CRC patients

	**All patients (N=271)**	**CD133 IHC expression (0)**	**CD133 IHC expression ** **(1-10%)**	**CD133 IHC expression ** **(11-50%)**	**CD133 IHC expression (51-100%)**
Variables	N	%	%	%	%
Gender					
Male	161	27.95	23.60	22.98	25.46
Female	110	32.72	20.00	20.00	27.27
Tumor site*					
Colon	150	37.33	22.66	17.33	17.33
Rectum	121	20.66	21.48	27.27	30.57
pTNM stage					
II	122	31.14	25.40	22.13	21.31
III	149	28.85	19.46	21.47	30.20
pT stage					
T1	4	75.00	0	0	25.00
T2	6	16.66	66.66	16.66	0
T3	239	29.70	21.75	22.17	26.35
T4	22	27.27	18.18	22.72	31.81
pN stage					
N0	123	30.90	25.20	21.95	21.95
N1	88	29.54	17.04	25.00	28.40
N2	56	26.78	25.00	17.85	30.35
N3	4	50.00	0	0	50.00
Histologic differentiation*					
Well	16	37.50	31.25	18.57	12.50
Moderate	225	25.33	22.22	22.66	29.77
Poor	15	53.33	20	26.66	0
Mucinous adenocarcinoma	15	66.66	13.33	6.66	13.33
Adjuvant therapy					
Yes	171	28.07	22.22	18.71	30.99
No	100	33.00	22.00	27.00	18.00

* p<0.05.

### CD133 mRNA expression

Available fresh frozen tumor tissues from 75 cancers (21 colon and 54 rectum) among 271 CRCs were used for real-time RT-PCR to measure CD133 mRNA expression. The mRNA expression was found to be significantly correlated with the CD133 IHC expression (Table 4) (p=0.0257).

**Table 4:** Cross-table showing correlation among CD133 IHC staining, mRNA expression and methylation level in CRC analyzed by Pearson correlation

		**mRNA**	**Promoter methylation**	IHC**
mRNA	Pearson correlation	1	-0.18539	0.25763
	p-value		0.1113	0.0257*
Promoter methylation	Pearson correlation	-0.18539	1	-0.38263
	p-value	0.1113		<0.0001*
IHC**	Pearson correlation	0.25763	-0.38263	1
	p-value	0.0257*	<0.0001*	

*p<0.05.

** Immunohistochemical stain.

### The correlation between CD133 expression and promoter methylation

CD133 promoters were more hypomethylated (Table 4) in cases with higher CD133 IHC expression, while hypermethylation was noted in cases with lower CD133 IHC expression. This inverse correlation between CD133 IHC expression and promoter methylation was statistically significant (p=0.0001). However, CD133 mRNA expression level was not significantly correlated with promoter methylation (p=0.1113).

### The prognostic significance of CD133 expression in CRC patients according to the adjuvant treatment

**Figure 3:**
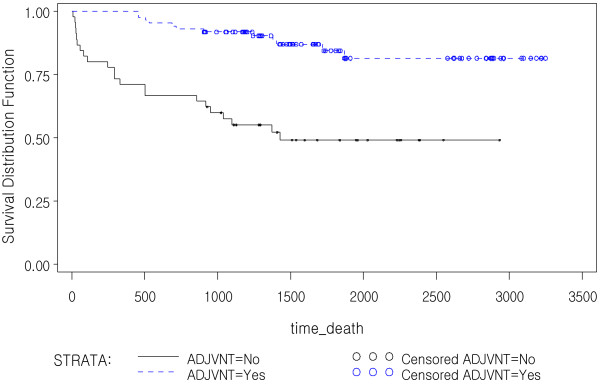
**Kaplan-Meier survival curves in the CD133+ (≥10% expression) colorectal cancer patients according to adjuvant therapy status.** The patients with CD133+ CRC had a significantly shorter OS if they had not received adjuvant therapy (p=0.0001).

**Figure 4:**
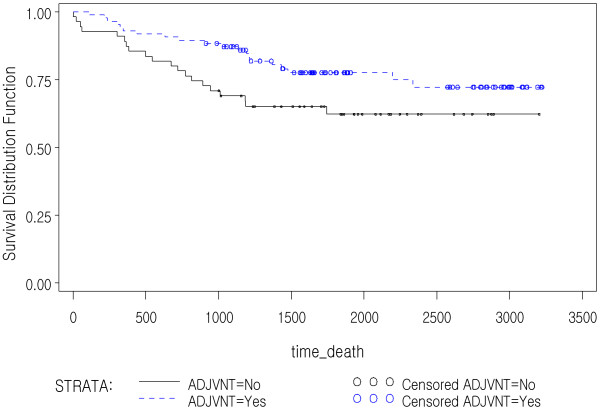
**Kaplan-Meier survival curves in the CD133- (<10% expression) colorectal cancer patients according to adjuvant therapy status.** There was no statistically significant difference in OS according to the adjuvant therapy status (p=0.055).

In multivariate analysis, CD133 IHC expression was not an independent prognostic factor in stage II and III colorectal cancer in this study. Patients receiving adjuvant therapy have a significantly longer OS time compared to those without adjuvant therapy (p<0.0001). And among the group with CD133+ tumors in this study (>10%, n=130), patients with adjuvant therapy (n=85) had a better OS compared to those without adjuvant therapy (n=45) (p<0.0001, HR 0.125, 95% CI 0.052-0.299, Figure 3). However, the CD133- tumors (≤10%, n=141) did not show significant difference between two groups (n=86 vs. 55, p=0.055, HR 0.500, 95% CI 0.247-1.015, Figure 4). There was no significant correlation between CD133 IHC expression and DFS according to adjuvant therapy (p=0.2451). CD133 mRNA also was not significantly correlated with patients’ survival (p=0.3186) or recurrence of tumors (p=0.3198) in Cox proportional regression test adjusted with age, stage, and adjuvant therapy. Due to limited number of cases with available fresh frozen tissue, we could not analyze the prognostic significance of mRNA expression according to adjuvant therapy.

## Discussion

The CSC theory finds a concrete basis of rationality in colorectal cancer owing to the fact that colon epithelium physiologically regenerates and is shed periodically over a short span of time not compatible with traditional model of carcinogenesis according to which cells ought to be more long-lived and suffer several mutations and genetic alterations to be able to convert to a cancer cell [32,33].

We described the distribution and prognostic significance of CSC marker, CD133 expression in 271 CRCs in this study. CD133 was expressed in 47.97% of CRCs. It is higher than previously reported. Previous studies have revealed controversial findings regarding the pattern (cytoplasmic vs. membranous) and distribution of CD133 IHC staining in CRCs. These differences in previous reports could arise from using different antibodies, tissue samples (cell lines vs. human tissue), methods of detection (IHC expression vs. PCR based techniques) [34], and tissue sampling method (tissue microarray vs. individually mounted tissue slides) and method for scoring the positivity of CD133 IHC expression. To avoid the scoring bias and in view of a recent paper in which CD133 positivity was quantitatively graded [35], we used four-tiered scoring method comprising 0 (totally negative cases), 1+, 2+, and 3+ cases (CD133+ cells covering 1-10%, 11-50%, and 51-100%, of the tumor area, respectively). Nevertheless, we considered the 2+ and 3+ groups as CD133 positive.

On the other hand, due to the fact that CD133 is not homogenously expressed and inability of microarray to fully represent the whole tumor, we used individually-mounted whole-block tissue slides for IHC analysis. Furthermore, to decrease the staining bias, we used automated machine for all procedure of IHC staining.

In line with findings of previous studies on colon [36-38] tumors with moderately differentiation showed higher level of CD133 IHC expression compared to poorly differentiated tumors and mucinous adenocarcinomas. No difference was noted in IHC expression between superficial and deep areas (Figure 2F). We rarely found unequivocal cytoplasmic or luminal staining at the crypt base in non-neoplastic colonic mucosa around the tumor, similar to the results of previous studies [20,26,39]. In comparison with the CD133 IHC expression of non-neoplastic colonic mucosa, there are more frequent and strong CD133 expression in the luminal border of non-neoplastic mucosa of stomach and pancreas even the reason is unknown. Given these results, further study seems to be required to clarify whether CD133 is a colon cancer stem cell marker or not.

In this study, we used monoclonal antibody against the CD133/1 or AC133, one of the two epitopes of the CD133 protein. The other epitope is AC141. Although, the monoclonal antibodies against these two epitopes have been interchangeably used to purify and characterize various stem and progenitor cells [40] there is rarely discordant expression of the AC133 and AC141 epitopes observed such as in a study on patients with myelodysplastic syndrome and acute myelogenous leukemia [41]. In addition, few important factors need to be considered while using monoclonal antibodies against an epitope of CD133. First of all, there is little known about the characteristics of the two epitopes detected by the monoclonal antibodies. Secondly, these epitopes are suggested to be glycosylated (however the supporting evidence for this claim is not well verified in the existing literature [42]) and this glycosylation is reported to be down-regulated upon differentiation of epithelial cells. An additional confusing factor is the presence of alternatively spliced variants of CD133. There in human CD133 gene exist at least 37 exons and several alternatively spliced forms [21]. Although, there is little knowledge about the existence of alternatively spliced CD133 isoforms that lack the AC133 or AC141 epitopes, the epitope-negative cells (cells that are negative against the monoclonal antibodies such as the CD133 negative cases in our study) may not solely and necessarily mean CD133 negativity in the absence of proper verification of CD133 protein or mRNA levels [42].

Additionally, it was recently concluded that AC133 does not recognize a glycosylated epitope, in contrast to previous suggestions [43,44] and described that differential splicing is also not the cause of differential AC133 recognition. However, it remains for the future studies to comparatively use antibodies against all known glycosylated and non-glycosylated epitopes of CD133 to draw a confident conclusion over the validity of the tested monoclonal antibodies.

To validate our IHC results in CRCs, we also evaluated CD133 mRNA expression in 75 cases out of 271 cases which had available fresh frozen tissue. There was a significant correlation between mRNA expression and CD133 IHC expression (p=0.0257). Since the CD133 IHC expression happen to be observed only in tumor (with the exception of rare cells in crypt base) and there is significant direct correlation between the IHC and mRNA expression level, there may be minute chance of missing isoforms of CD133 (if any) that may lack epitope-immunoreactivity via our IHC staining. Unfortunately, we could not evaluate the prognostic significance of CD133 mRNA expression according to the adjuvant therapy status due to limitation in number of cases with available fresh frozen tissue.

To verify the regulatory mechanism of CD133 expression, we performed methylation analysis and found inverse correlation between CD133 expression and promoter methylation level (p<0.0001). This finding is concordant with previous study on colon cancer cell lines [31]. But, the correlation of CD133 mRNA with methylation was not statistically significant. The lack of statistical significance in correlation between the level of CD133 mRNA and promoter methylation suggests that other factors may be additionally involved in the regulation of CD133 expression.

We studied the correlation between CD133 IHC expression and patients’ survival in stage II and III CRCs. Although CD133 IHC expression was not correlated with OS (p=0.9778) and DFS (p=0.2451), the group of patients with CD133+ CRC showed better OS if patients received adjuvant therapy compared to patients without adjuvant therapy in the Log-Rank test. Multivariate analysis adjusted with age and stage also showed statistical significance between two groups (p<0.0001, HR 0.125, 95% CI 0.052-0.299). However the patients with CD133- tumors did not show any difference in OS between two groups (p=0.055, HR 0.500, 95% CI 0.247-1.015). Therefore the adjuvant therapy can be of benefit for patients with CD133+ tumor in contrast to patients with CD133- one. This stands against the notion that tumors with high CD133 positivity are resistant to adjuvant therapy [39]. Our results are in support of a recent paper which has demonstrated that CD133+ tumor cells are not more resistant to chemotherapy than CD133- tumor cells [28]. Noteworthily, this finding asks for further elucidation of the matter and moreover notifies that stage II and III colon cancer patients with CD133 IHC expression may benefit from adjuvant therapy. However, adjuvant therapy status seemed not to have affected DFS in patients with CD133+ as well as CD133- tumors. Our finding on the one hand questions the non-response to chemotherapy theory and on the other hand asks for further elucidation of the precise prognostic role of CD133 as an important prognostic factor for considering adjuvant therapy in stage II and III colon cancer. Future cohort studies with more number of patients in the two groups according to adjuvant therapy may further enlighten this finding.

## Conclusion

In conclusion, CD133 expression in CRCs may be regulated by promoter methylation and CD133 IHC expression notifies a better prognosis in stage II and III CRC patients who have adjuvant therapy. However CD133 IHC expression is not an independent prognostic factor in patients with stage II and III CRC.

## Competing interests

The authors have declared that no competing interests exist.

## Authors’ contribution

KMJ developed the research proposal and collected data, assisted in reviewing the slides, and wrote the draft manuscript. SYJ performed molecular analysis. IYK collected the clinical data and wrote the clinical part of method. SSO worked for the preliminary data analysis. EHC completed the statistical analysis and making the figures. SJJ contributed in statistical analysis. TYK performed immunohistochemical stain. MYC designed and supervised this research and finalized the manuscript. All authors have read and approved the final manuscript.

## Pre-publication history

The pre-publication history for this paper can be accessed here:

http://www.biomedcentral.com/1471-2407/13/166/prepub
